# Enhancing second harmonic generation by Q-boosting lossless cavities beyond the time bandwidth limit

**DOI:** 10.1515/nanoph-2023-0389

**Published:** 2024-01-02

**Authors:** Paolo Franceschini, Andrea Tognazzi, Anna M. Chernyak, Alexander I. Musorin, Alfonso C. Cino, Andrey A. Fedyanin, Costantino De Angelis

**Affiliations:** Department of Information Engineering, University of Brescia, Via Branze 38, 25123, Brescia, Italy; National Institute of Optics – National Research Council (INO-CNR), Via Branze 45, 25123, Brescia, Italy; Department of Engineering, University of Palermo, Viale delle Scienze ed. 9, 90128, Palermo, Italy; Faculty of Physics, Lomonosov Moscow State University, Leninskie gory 1, 119991, Moscow, Russia

**Keywords:** time-bandwidth limit, second harmonic generation, time-varying metasurface, *Q*-boosting

## Abstract

Nanostructures proved to be versatile platforms to control the electromagnetic field at subwavelength scale. Indeed, high-quality-factors nanocavities have been used to boost and control nonlinear frequency generation by increasing the light–matter interaction. However, nonlinear processes are triggered by high-intensities, which are provided by ultrashort laser pulses with large bandwidth, which cannot be fully exploited in such devices. Time-varying optical systems allow one to overcome the time-bandwidth limit by modulating the cavity external coupling. Here we present a general treatment, based on coupled mode theory, to describe second harmonic generation in a doubly resonant cavity for which the quality-factor at the fundamental frequency is modulated in time. We identify the initial quality factor maximizing second harmonic efficiency when performing *Q*-boosting and we predict a theoretical energy conversion efficiency close to unity. Our results have direct impact on the design of next generation time-dependent metasurfaces to boost nonlinear frequency conversion of ultrashort laser pulses.

## 1 Introduction

Nonlinear optics has a variety of applications ranging from medicine to communication and laser frequency conversion [1–3]. The pump electric field intensity is one of the key factors determining nonlinear processes efficiency; thus, ultrashort laser pulses are often used, since they feature high peak intensities. Dielectric nanostructures represent a compact and versatile solution to control the phase, polarization, intensity, and electric field distribution on a subwavelength scale, broadening the functionalities offered by bulky crystals and down-scaling the device dimensions [4–10]. However, resonant structures are subject to the so-called time-bandwidth limitation, *i.e.*, the acceptance bandwidth of the device is ≈ω/Q , where *ω* is the resonant radial frequency and *Q* is the quality-factor (*Q*-factor) [11]. Indeed, single resonators with low *Q*-factors (*Q* < 50) have short light–matter interaction time, which results in limited nonlinear conversion efficiency. On the other hand, metasurfaces and photonic crystals have larger *Q*-factors (*Q* > 200), which correspond to longer in-cavity lifetimes, but does not allow to couple all the incoming laser pulse spectrum to the device, ultimately limiting the performances. Overcoming the time-bandwidth limit is of paramount importance to fully exploit high-*Q* photonic devices, not only for nonlinear frequency conversion but also with any broadband radiation source. Previously, excitation with complex frequencies has been proposed to enhance coupling between the incoming radiation and the cavity [12, 13]. However, it is hard to sustain an exponentially increasing field amplitude profile over long time periods. In this framework, the possibility to tune the optical properties of metasurfaces with unprecedented speed, as fast as hundreds of fs [14–20], paved the way to devices operating beyond such limitation.

Time-variant metasurfaces allow to overcome the time-bandwidth tradeoff and extend metasurfaces functionalities [21–23]. Indeed recently, pulse generation and compression [24], coupling beyond the time bandwidth limit [25–28], spectral bandwidth manipulation [29–33], enhanced frequency conversion [34, 35] and light-storage [36, 37] were demonstrated. Coupled mode theory (CMT) represents a widespread tool to describe time-varying resonators [28, 38] and nonlinearities [39], since it can be applied from radio to optical frequencies. Although, CMT has been successfully applied to describe third harmonic generation at optical frequencies with time dependent *Q*-factors at the fundamental frequency (FF) [35], second harmonic generation (SHG) and the coupling between the fundamental and higher harmonic mode have yet to be considered. The underlying idea of this work, to efficiently transfer energy from FF to SH mode, is to couple the incoming laser pulse to a low-*Q* cavity and to modulate its *Q*-factor to increase the energy stored inside the resonator. This leads to an increased second harmonic (SH) conversion efficiency.

Here, we focus on doubly resonant cavities featuring an internal coupling between the modes at the fundamental and at its SH frequency. We provide a general treatment based on CMT (including also pump-depletion effects) to boost SHG of light pulses by modulating the external cavity coupling term at FF in a lossless doubly resonant cavity excited by an input pulse at FF. First, we extend the CMT model for SHG in Ref. [40] to a time-dependent doubly resonant cavity. Then, we study the role of time delay *τ*, *i.e.*, the time interval between the external pulse arrival and the instant at which the switching occurs. After that, we investigate the effect of the amplitude modulation of the *Q*-factor at the FF (before and after the switching) and compare our results with the static case, *i.e.*, when *Q*-factor is constant over time. We identify the initial and final *Q*-factor at the FF, and their relation with the external pulse duration, which maximize SH conversion. We demonstrate that the initial *Q*-factor plays a major role in boosting SHG. Our results pave the way to a deeper understanding of metasurfaces operating beyond the time bandwidth limit to boost nonlinear frequency conversion exploiting *Q*-boosting.

## 2 Results

### 2.1 Coupled mode theory for doubly-resonant second harmonic cavities

**Figure 1:**
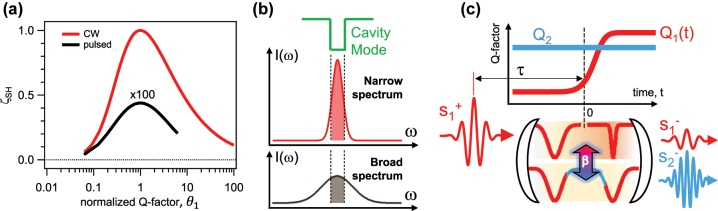
Framework and design of Q-boosting approach. (a) Static case second harmonic power conversion efficiency (*ζ*_*SH*_) as a function of the normalized *Q*-factor $\left(\theta_{1}=Q_{1}/Q_{1}^{\text{opt}}\right)$ for a continuous-wave (CW, red curve) and pulsed (black curve, factor × 100 magnification) input sources $s_{1}^{+}$ . For both CW and pulsed excitation cases, an optimal set of the cavity parameters exists (see Section 1 of the Supplementary Material). For both cases, quality factor of mode 2 (*Q*_2_) is 1500. The value of internal coupling (*β*_1_) is 1.5 × 10^−4^ $\sqrt{1\text{/J}}$ for the CW case and 1.5 × 10^−4^ $\sqrt{\text{fs/J}}$ for the pulsed case. (b) Amount of spectral power (shaded region) efficiently stored in a cavity mode (green line) for a narrow (red, top panel) and broadband pulse (black, bottom panel). (c) Time-dependent amplitude modulation (Equation (3)) of the *Q*-factor at *ω*_1_ (*Q*_1_) to enhance second harmonic conversion efficiency. The $s_{1}^{+}$ pulse resonantly excites the cavity mode at *ω*_1_ (bottom panel). Given the coupling (*β*) between the cavity modes, by carefully tuning the temporal delay (*τ*) between the arrival of the pulse $s_{1}^{+}$ and the time at which the *Q*_1_ modulation occurs, the relative amplitude of the outgoing waves at *ω*_1_ $\left(s_{1}^{-}\right)$ and *ω*_2_ = 2*ω*_1_ $\left(s_{2}^{-}\right)$ can be controlled.

Here, we employ CMT to describe SHG in a doubly-resonant cavity, which supports modes at both FF, *ω*_1_, and SH frequency, *ω*_2_ = 2*ω*_1_, with amplitudes *a*_1_ and *a*_2_, respectively [41]. The cavity is excited by an external source $s_{1}^{+}$ , at frequency *ω*_*p*_ = *ω*_1_ (resonant excitation). In general, the set of equations describing SHG in a doubly-resonant cavity is [40, 42]:

(1) $$\left\{\begin{array}{cc} \frac{\text{d}a_{1}}{\text{d}t} & =\omega_{1}\left(i-\frac{1}{2Q_{1}}\right)a_{1}-i\omega_{1}\;\beta_{1}a_{1}^{*}a_{2}+\sqrt{\frac{\omega_{1}}{Q_{1}}}\;s_{1}^{+}\\ \frac{\text{d}a_{2}}{\text{d}t} & =\omega_{2}\left(i-\frac{1}{2Q_{2}}\right)a_{2}-i\omega_{2}\beta_{2}a_{1}^{2}\\ s_{1}^{-} & =-s_{1}^{+}+\sqrt{\frac{\omega_{1}}{Q_{1}}}\;a_{1}\\ s_{2}^{-} & =\sqrt{\frac{\omega_{2}}{Q_{2}}}\;a_{2} \end{array}\right.,$$

where *Q*_1_ and *Q*_2_ are the *Q*-factors of modes 1 (FF) and 2 (SH), *β*_1_ and *β*_2_ are the internal cavity coupling coefficients, which are responsible for pump depletion and SH conversion, respectively, and they are related to the overlap integral between the FF and SH modes [40, 43]. As pointed out in Ref. [40], we set *β*_2_ = *β*_1_/2 to fulfill the conservation energy constraint. The terms $s_{1}^{-}$ and $s_{2}^{-}$ represent the outgoing waves at *ω*_1_ and *ω*_2_, respectively. Although not being explicitly reported, time dependence of *a*_*k*_ and $s_{k}^{\pm }$ terms in Equation (1) is implied. In analogy with [40], we define the SH (FF) power conversion efficiency as $\zeta_{SH}=P_{2}^{-}/P_{1}^{+}$ $\left(\zeta_{FF}=P_{1}^{-}/P_{1}^{+}\right)$ , where $P_{2}^{-}$ $\left(P_{1}^{-}\right)$ is the output power at *ω*_2_ (*ω*_1_) and $P_{1}^{+}$ is the input power at *ω*_*p*_ = *ω*_1_. First, we focus on the SH power conversion within a cavity where *Q*_1_ is constant over time (see Figure 1a). When the input source is a monochromatic continuous-plane-wave (CW), complete power conversion efficiency ( $\zeta_{SH}={\left|s_{2,ss}^{-}\right|}^{2}/{\left|s_{1}^{+}\right|}^{2}=1$ [40]) can be achieved for an optimum value of *Q*_1_ $\left(Q_{1}^{\text{opt}}\right)$ in the steady state (ss) condition [40]. As an example, we calculate *ζ*_*SH*_ for various values of *Q*_1_ by solving Equation (1) and we display the results as a function of the normalized *Q*-factor $\left(\theta_{1}=Q_{1}/Q_{1}^{\text{opt}}\right)$ in Figure 1a (red curve) for the following set of parameters: *ω*_1_ = 0.791 rad/fs (*ν* = 125.9 THz), *β*_1_ = 1.5 × 10^−4^ $\sqrt{1\text{/J}}$ , *Q*_2_ = 1500, and $\left|s_{1}^{+}\right|=17.33$ $\sqrt{\text{W}}$ . The value of $Q_{1}^{\text{opt}}$ is bounded to *ω*_1_, *β*_1_, *Q*_2_ and $|s_{1}^{+}{|}^{2}$ [40] (see Section 1.1 of the Supplementary Material for more details).

Now, we consider a pulsed input source at resonance (*ω*_*p*_ = *ω*_1_) with Gaussian temporal profile of the form:

(2) $$s_{1}^{+}\left(t\right)=s_{0}\cdot \sqrt[{4}]{\left(4\ln2\right)/\left(\pi \tau_{p}^{2}\right)}\cdot {\text{e}}^{-\frac{2\ln2\;t^{2}}{\tau_{p}^{2}}}\cdot {\text{e}}^{\text{i}\omega_{p}t},$$

where *τ*_*p*_ is the full width at half maximum (FWHM) of the time-dependent intensity profile $I\;\left(t\right)\propto {\left|s_{1}^{+}\;\left(t\right)\right|}^{2}$ and *s*_0_ is the amplitude. $s_{1}^{+}\;\left(t\right)$ and $s_{k}^{-}\;\left(t\right)$ are the time-dependent amplitudes of the input and output waves, respectively [41]. In analogy with [34, 35], the quantity $U_{k}=\int_{-\infty }^{+\infty }{\left|a_{k}\;\left(t\right)\right|}^{2}\;\text{d}t$ defines the time-integrated energy (units [J s]) inside the cavity ascribed to the mode *k* and the pulse energy of the incoming and outgoing radiation (units [J]) are defined as $V_{1}^{+}=\int_{-\infty }^{+\infty }{\left|s_{1}^{+}\;\left(t\right)\right|}^{2}\;\text{d}t=s_{0}^{2}$ and $V_{k}^{-}=\int_{-\infty }^{+\infty }{\left|s_{k}^{-}\;\left(t\right)\right|}^{2}\;\text{d}t$ , respectively. Consequently, the input and output pulse power is calculated as the ratio between the pulse energy and its temporal duration: $P_{k}^{\pm }\;=\;V_{k}^{\pm }/\Delta t_{k}^{\pm }$ (see Supplementary Material for more details). For a pulsed source, the maximum achievable *ζ*_*SH*_ $\left(\zeta_{SH}^{\max}\right)$ is much smaller than in the CW case. As shown by the black curve in Figure 1a, for *τ*_*p*_ = 100 fs, *β*_1_ = 1.5 × 10^−4^ $\sqrt{\text{fs/J}}$ and *s*_0_ = 17.33 $\sqrt{\text{J}}$ , $\zeta_{SH}^{\max}$ ≃ 0.44 % (see Supplementary Material). The difference with the monochromatic CW can be rationalized by recalling that, for a pulsed excitation, the spectral intensity profile (*S*(*ω*)) has a bandwidth Δ*ω*_*p*_, which is related to the transform-limited pulse duration *τ*_*p*_ by the time-bandwidth product: *τ*_*p*_ ⋅ Δ*ω*_*p*_ = 4ln2. Therefore, only the fraction of $S\;\left(\omega \right)$ matching the bandwidth of the cavity mode (shaded region in Figure 1b) is involved in the SH conversion process. A *Q*-boosting approach allow to increase the energy stored inside the cavity by a dynamic control of the *Q*-factor.

### 2.2 Time dependent *Q*-factor modulation

In Figure 1c, we depict the working principle of *Q*-boosting to enhance SHG. We consider a time-dependent amplitude modulation of *Q*_1_ (occurring at *t* = 0), which, in the following part of the work, takes the form

(3) $$\frac{1}{Q_{1}\left(t\right)}=\frac{1}{Q_{1L}}+\frac{1}{2}\left(\frac{1}{Q_{1H}}-\frac{1}{Q_{1L}}\right)\left[1+\text{erf}\left(\frac{t}{\sigma }\right)\right],$$

where *σ* is the switching time, *Q*_1*L*_ and *Q*_1*H*_ being the initial and final values of the FF *Q*-factor, respectively. In our model, the variation of *Q* corresponds to the change in the mode bandwidth *γ*, *i.e.*, *Q*_1_(*t*) = *ω*_1_/(2*γ*_1_(*t*)). Moreover, as sketched in Figure 1c, we allow the input pulse $s_{1}^{+}$ to enter the cavity with a delay time *τ* with respect to the instant at which *Q*_1_ increases. Therefore, from Equation (2), the expression for $s_{1}^{+}$ becomes $s_{1}^{+}\left(t;\tau \right)=s_{1}^{+}\left(t-\tau \right)$ . Here, the value of *Q*_2_ is constant over time and, in the following, we assume *Q*_2_ = 1500. In general, *β*_1_ should change as the electric field distribution inside the cavity varies. Here, we assume those modifications to be negligible and consider *β*_1_ to be constant over time.

**Figure 2:**
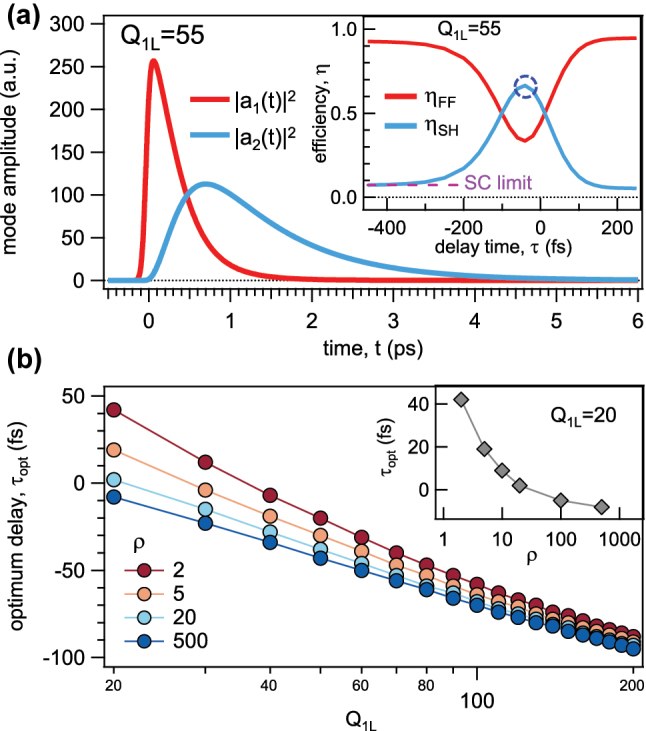
Coupled mode theory results within Q-boosted doubly resonant cavities. (a) Squared modulus of the fundamental frequency (FF) mode |*a*_1_|^2^ (red curve) and squared modulus of the second harmonic (SH) mode |*a*_2_|^2^ (blue curve) as a function of time *t* for *τ* = −40 fs. The inset reports the FF (red curve) and SH (blue curve) conversion efficiency (*η*_*FF*_ and *η*_*SH*_, respectively) as a function of the temporal delay *τ*. The blue circle highlights the optimum delay *τ* ^opt^ ≈ − 40 fs case. SC: static case. (b) *τ* ^opt^ as a function of the initial quality-factor *Q*_1*L*_ for different ratios *ρ* = *Q*_1*H*_/*Q*_1*L*_, where *Q*_1*H*_ is the final quality-factor. The inset reports *τ* ^opt^ as a function of *ρ* for *Q*_1*L*_ = 20.

First, we numerically solve Equation (1), endowed by the time-dependent *Q*_1_ as in Equation (3) and assuming *Q*_1*L*_ = 55 and *σ* = 50 fs. We introduce the modulation amplitude as *ρ* = *Q*_1*H*_/*Q*_1*L*_. The numerical results obtained for *ρ* = 20 are displayed in Figure 2a, which shows the temporal dynamics of the modes amplitude ${\left|a_{1}\left(t\right)\right|}^{2}$ and ${\left|a_{2}\left(t\right)\right|}^{2}$ (red and blue curve, respectively), excited by an input pulse with duration *τ*_*p*_ = 100 fs and entering the cavity at *τ* = −40 fs. The value of |*a*_*k*_(*t*)|^2^ (*k* = 1, 2) is related to the instantaneous energy accumulated in the mode *k* [41] and decreases exponentially over time due to the *ω*_*k*_/(2*Q*_*k*_)*a*_*k*_ term in Equation (1). Here, we introduce the SH energy conversion efficiency as $\eta_{SH}=V_{2}^{-}/V_{1}^{+}$ . At this stage it is important to underline that *η*_*SH*_ is related to *ζ*_*SH*_ through the term $\tau_{p}/\Delta t_{2}^{-}$ and the two frameworks allow to extract the same information (see Section 3 of the Supplementary Material). Therefore, we prefer to present the results in terms of *η*_*SH*_. As displayed in the inset in Figure 2a, the optimum delay time (*τ*_opt_), which maximizes the SH conversion efficiency, is *τ*_opt_ ≈ −40 fs (blue circle) and it is of the order of the resonator lifetime *Q*_1*L*_/*ω*_1_ ≈ 70 fs, consistent with [35]. Moreover, the inset in Figure 2a shows that, for *τ* ≪ −*τ*_*p*_, *i.e.*, the cavity has not switched yet, *η*_*SH*_ is the same as in the static case (SC, *η*_*SH*_ ∼ 7 %) when *Q*_1_ = *Q*_1*L*_ (dashed purple line in Figure 2a, inset). On the other hand, for *τ* ≫ *τ*_*p*_, the input pulse enters the cavity when the switching has already occurred, thus the pulse essentially couples to a high-*Q* cavity, resulting in low *η*_*SH*_ (since a large portion of the incoming spectrum falls outside the cavity acceptance bandwidth). When −*τ*_*p*_ ≲ *τ* ≲ *τ*_*p*_, a large *η*_*SH*_ enhancement is achieved, since the radiation channel at the fundamental frequency reduces after a large portion of the incoming energy pulse has coupled to the cavity. In Figure 2a, *η*_*SH*_ increases from ∼7 % to ∼67 %. As expected, $\eta_{FF}=V_{1}^{-}/V_{1}^{+}$ decreases when *η*_*SH*_ increases.

Now, we discuss how *τ*_opt_ relates to *ρ*. In Figure 2b, we report *τ* ^opt^ as a function of *Q*_1*L*_ for different *Q*-factor ratios *ρ* = *Q*_1*H*_/*Q*_1*L*_ with *τ*_*p*_ = 100 fs (see Section 2 of the Supplementary Material for more details). For *ρ* ≲ 20 and *Q*_1*L*_ ≲ 30, *τ* ^opt^ is positive, meaning that the switch should occur before the pulse enters the cavity. Moreover, for small *Q*_1*L*_, *τ* ^opt^ strongly depends on *ρ*, while for large *Q*_1*L*_, *τ* ^opt^ becomes almost independent from *ρ* (see the inset in Figure 2b). We note that as *Q*_1*L*_ increases, *τ* ^opt^ becomes negative for all values of *ρ*, meaning that the switching should occur once the pulse is already inside the cavity. The inset in Figure 2b shows that, for a fixed *Q*_1*L*_, *τ* is critical only for small values of *ρ*. This is of particular interest since, in practice, small *ρ* are easier to induce and different ways to dynamically manipulate a cavity *Q*-factor have already been proposed [15, 16, 35, 44]. However, we stress that our model can be applied to a wider class of doubly resonant cavities beyond metasurfaces. Therefore, the results shown in Figure 2b allow identifying the relevant timescale regarding the tuning of the delay time *τ*.

In the following, we focus only on the dependence of SH conversion efficiency upon *Q*_1*L*_ and *Q*_1*H*_, since a more detailed analysis of the exact dependence of *τ* ^opt^ on the other relevant parameters is beyond the scope of the present work. We calculate the optimum values of *Q*_1*L*_ and *Q*_1*H*_, denoted as $Q_{1L}^{\text{opt}}$ and $Q_{1H}^{\text{opt}}$ , which maximize *η*_*SH*_, by computing:

$$\underset{\tau }{\max}\;\eta_{SH}\left(Q_{1L},Q_{1H},\tau \right).$$

**Figure 3:**
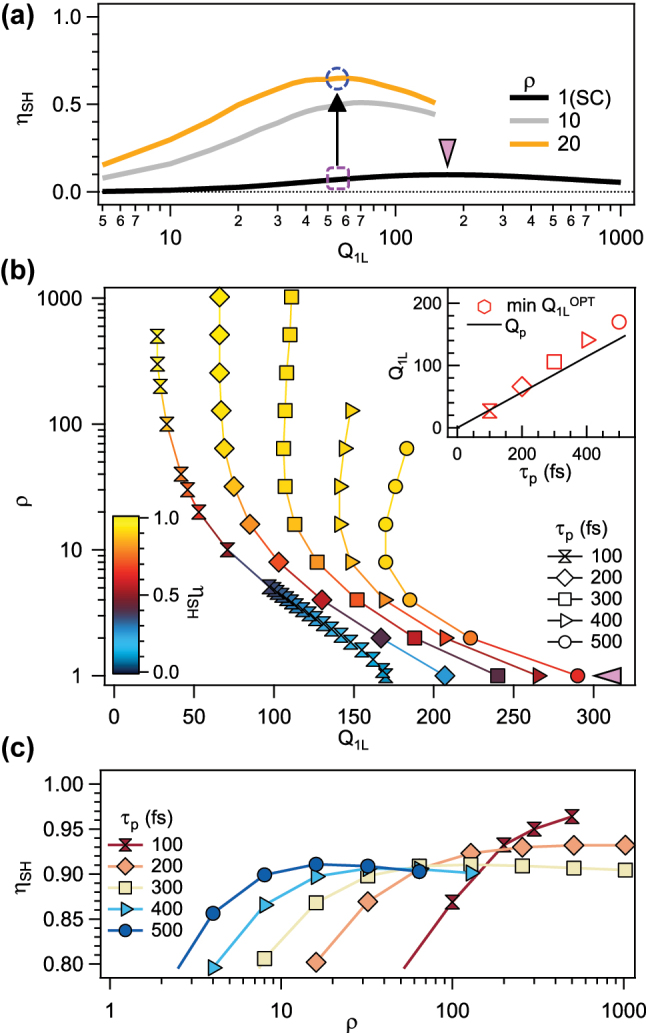
Optimal configurations for efficiency enhancement. (a) Second harmonic conversion efficiency (*η*_*SH*_) at the optimum delay time (*τ* ^opt^) as a function of the initial quality-factor (*Q*_1*L*_) for different values of *ρ* = *Q*_1*H*_/*Q*_1*L*_, where *Q*_1*H*_ is the final *Q*-factor: 1 (black curve, static case), 10 (gray curve), and 20 (yellow curve). The black vertical arrow highlights the *η*_*SH*_ enhancement for *Q*_1*L*_ = 55 (case displayed in Figure 2a). (b) Combinations of *Q*_1*L*_ and *ρ* at *τ* ^opt^ leading to maximum *η*_*SH*_, for different impinging pulse duration (*τ*_*p*_). The value of *η*_*SH*_ is indicated by the colorscale. The inset reports the *Q*_1*L*_ corresponding to maximum *η*_*SH*_ as a function of *τ*_*p*_. The black line corresponds to the bandwidth-matching condition *ω*_1_/(*Q*_1*L*_) = 1/*τ*_*p*_. (c) *η*_*SH*_ as a function of *ρ* for different *τ*_*p*_. For each *ρ* ^opt^, it is possible to extract the corresponding $Q_{1L}^{\text{opt}}$ from panel (b). In the panels, the pink triangles indicates the maximum conversion efficiency in the static case (*ρ* = 1).

In Figure 3a, we report *η*_*SH*_ as a function of *Q*_1*L*_ for different *ρ* values. The black curve corresponds to the static case (*Q*_1_ = *Q*_1*L*_ = *Q*_1*H*_ → *ρ* = 1) and the purple triangle highlights the maximum SH conversion efficiency $\left(\eta_{SH}^{\max}\right)$ for the static case. We note that, at constant *Q*_1*L*_, *η*_*SH*_ increases with increasing *ρ*. Indeed, an increasing *Q*_1*H*_ value corresponds to a decrease of the radiative losses of the FF mode; thus, the energy transferred from the FF to the SH mode (through the internal coupling term *β*_2_) increases. The vertical arrow highlights what happens in a *Q*-boosted cavity, *i.e.*, fixed *τ*_*p*_ and *ω*_1_, for *Q*_1*L*_ = 55 (reference case in Figure 2a). A suitable choice of *Q*_1*L*_ and *Q*_1*H*_ values allows to enhance *η*_*SH*_ (blue circle) compared to the static case (violet square). The maximum conversion efficiency is attained for a *Q*_1*L*_ which is different from the optimal *Q*_1_ in the static case. We also note that $Q_{1L}^{\text{opt}}$ decreases as *ρ* increases.

In order to formulate useful guidelines for time-dependent doubly resonant cavities operation, we report in Figure 3b the trajectories (for various *τ*_*p*_ values), in the plane spanned by *Q*_1*L*_ and *ρ*, allowing a SH efficiency enhancement. Within a curve, each point represents the value of *Q*_1*L*_ which maximizes *η*_*SH*_ (*i.e.*, $Q_{1L}^{\text{opt}}$ ) at a given *ρ* and the colorscale highlights the corresponding SH efficiency value. At fixed *τ*_*p*_, $Q_{1L}^{\text{opt}}$ gradually shifts from the initial static case (*ρ* = 1, purple triangle) toward lower values and reaches, for increasing *ρ*, a minimum value $Q_{1L}^{\min}$ , which corresponds to $\eta_{SH}^{\max}$ (see Figure 3c). From Figure 3b inset, we note that $Q_{1L}^{\min}∼Q_{p}=\omega_{p}\;\tau_{p}/\left(4\;\ln2\right)$ , where *Q*_*p*_ is an effective *Q*-factor given by the parameters describing the control pulse; therefore, $Q_{1L}^{\min}$ is close, but not equal, to the bandwidth-matching condition.

In order to qualitatively explain the relation between $Q_{1L}^{\min}$ and *Q*_*p*_, we have to briefly discuss the properties of the spectral profile of the entities involved (see Section 3 of the Supplementary Material for more details). In the frequency domain, the spectral lineshape of the excited-wave amplitude within a resonant lossy system (at FF mode) is given by a Lorentzian function [25]

(4) $${L\;}_{FF}\;\left(\omega ;\Gamma \right)=\frac{1}{\pi }\cdot \frac{{\left(\Gamma /2\right)}^{2}}{{\left(\Gamma /2\right)}^{2}+{\left(\omega -\omega_{1}\right)}^{2}},$$

where Γ represents the spectral linewidth (FWHM). On the other hand, given the choice of the temporal profile of the control pulse in Equation (2), its spectral shape is given by a Gaussian function:

(5) $$G\;\left(\omega \right)=\sqrt{\frac{4\;\ln2}{\pi \;\Delta \omega_{p}^{2}}}\;\exp\left[-\frac{4\ln2}{\Delta \omega_{p}^{2}}\cdot {\left(\omega -\omega_{1}\right)}^{2}\right].$$

The SH conversion process involves an effective energy transfer from the input pulse $\left(s_{1}^{+}\right)$ to the FF mode via the external coupling coefficient and then a transfer to the SH mode through the internal coupling term *β*. To do so efficiently, it is required to suitably match the spectral energy, within a certain spectral interval (delimited by *ω*_*A*_ and *ω*_*B*_), of the input pulse and the FF mode. The optimal matching condition between $L_{FF}$ and G , centered at *ω*_1_, is given by:

(6) $$\overset{\omega_{B}}{\underset{\omega_{A}}{\int }}\text{d}\omega \;G\;\left(\omega \right)=\overset{\omega_{B}}{\underset{\omega_{A}}{\int }}\text{d}\omega \;L_{FF}\;\left(\omega ;\tilde{\Gamma }\right)$$

with *ω*_*A*,*B*_ = *ω*_1_ ∓ Δ*ω*_*p*_, which leads to $\tilde{\Gamma }/\Delta \omega_{p}≃0.4<1$ (see Section 3 of the Supplementary Material). Thus, the matched cavity bandwidth $\tilde{\Gamma }$ satisfies the relation: $\tilde{\Gamma }<\Delta \omega_{p}$ .

From Figure 3b, we note that the *ρ* value at which $Q_{1L}^{\min}$ occurs decreases with increasing *τ*_*p*_, suggesting that, for long *τ*_*p*_ values, *ρ* ≃ 1. This is consistent with the results obtained for the monochromatic CW case [40] (corresponding to *τ*_*p*_ → ∞), for which a complete SH conversion efficiency is observed in the steady state regime of constant *Q*-factor (*ρ* = 1).

As noted in Figure 3c, *η*_*SH*_ decreases for *ρ* > *ρ* ^opt^. This means that, after reaching *ρ* ^opt^, a further increase of the cavity acceptance bandwidth is of little use, since the spectral weight of the frequencies far from *ω*_1_ is negligible. Ideally, despite the pump depletion term (*β*_1_) is included, for an infinite value of *Q*_1*H*_ and very long time, all the radiation stored in *a*_1_ can only escape through mode *a*_2_. This is because increasing *Q*_1*H*_ corresponds to closing the only radiation channel of mode *a*_1_, thus the energy can only escape the cavity through frequency conversion to *ω*_2_, since the radiation channel of mode *a*_2_ remains open. As expected from energy conservation *η*_*SH*_ < 1 and significantly increases for shorter pulses. It is clear that *ρ* ^opt^ decreases as *τ*_*p*_ becomes longer.

### 2.3 Limitations and implementation strategies

The modulation of the external coupling parameter *Q*_1_ may have consequences from a spectral point of view, thus potentially limiting the validity of the model. Indeed, an abrupt amplitude modulation of the *Q*-factor might: (*i*) introduce additional components in the spectrum of *a*_1_ (*a*_2_) located far from the central frequency *ω*_1_ (*ω*_2_) in the neighborhood of *ω*_2_ (*ω*_1_) or (*ii*) induce a significant frequency shift of the modes. Regarding the former aspect, the most delicate point is related to the switching time parameter *σ*, *i.e.*, the speed at which the variation of *Q*_1_ occurs, compared to the optical cycle of the input pulse 2*π*/*ω*_1_. In our work, *σ* = 50 fs and *τ*_*p*_ = 100 fs. This means that, given the *Q*-factor dynamics in Equation (3), the time interval required to go from 10 % to 90 % of the entire variation is ∼70 fs, which is nearly 10 times larger than 2*π*/*ω*_1_ ∼ 7.9 fs. Therefore, although *σ* and *τ*_*p*_ are comparable, in the time interval 2*π*/*ω*_1_, the pulse intensity and the *Q*-factor can be assumed as constant. Regarding the latter aspect, to limit the impact of frequency shift in Q-boosting, specific conditions in refractive index and loss changes can be adopted in practical realizations [32], as demonstrated by FDTD simulations in Ref. [35]. The validity of our approach is further confirmed by continuous wavelet analysis of the modes dynamics [27], which reveal that the modes have a constant central frequency (see Section S5 of Supplementary Material).

For the effective implementation of this scheme in the case of broadband pulses, a large variation between the initial and final values of the cavity *Q*-factor is required to obtain a high SH conversion efficiency, as previously discussed. The practical realization of this can be achieved by the suitable design of a cavity endowed by a material-dependent sharp resonance, *e.g.*, a quasi-BIC, at fundamental frequency. Thanks to a control light pulse, a variation of the material dielectric function [45, 46] can be induced, which in turn causes a large modulation of the *Q*-factor amplitude at FF with small frequency shift. A model including large frequency shift of the fundamental and/or SH modes is beyond the scope of this work. In order to avoid two-photon absorption and free carrier generation effects, an external control pulse with energy smaller than half of the bandgap may be used as in LiNbO_3_ and other large bandgap materials with non-zero *χ* ^(2)^ [47]. Further theoretical development may take into account the role of non-radiative losses which may arise from free-carrier excitation or two-photon absorption. Other suitable platforms include dielectric slabs featuring a BIC mode which can be coupled to external radiation by means of a transient grating [48]. In such platforms the *Q*-factor can be dramatically increased in a short time by removing the transient grating, thus reducing any possible non-radiative loss and at the same time achieving a long-lived high-*Q* resonant condition.

## 3 Conclusions

To conclude, we have unveiled how to maximize SH efficiency in a *Q*-boosted doubly resonant cavity. First, by applying CMT, we have compared SHG for static and modulated *Q*-factors, and predicted that *η*_*SH*_ can be significantly increased in time-dependent cavities. Secondly, we have shown that the first parameter to be optimized should be the delay time, which must be carefully chosen depending on the initial and final *Q*-factors. In particular, the optimum delay goes from positive to negative values when *Q*_1*H*_/*Q*_1*L*_ increases. For proper delay time and *Q*_1*H*_/*Q*_1*L*_, we have predicted SH efficiency conversion close to unity.

We have shown that perfect bandwidth-matching between cavity and pulse cannot be achieved due to different lineshapes. Thus, $Q_{1L}^{\text{opt}}$ is the one maximizing the overlap between a Gaussian and a Lorentizan. Additionally, we have found that the maximum conversion efficiency is not achieved when the cavity radiative losses at the fundamental becomes zero, *i.e.*, *Q*_1*H*_ → +∞. Instead, its value is determined by a compromise between a lower value, allowing a full collection of photons inside the cavity, and a longer cavity lifetime. Moreover, as the pulse duration increases, $Q_{1H}^{\text{opt}}$ and $Q_{1L}^{\text{opt}}$ get closer values; ultimately leading to the CW static case when *τ*_*p*_ → +∞.

Here, we have shown that both low-*Q* and high-*Q* resonators benefit from this approach. The formulated guidelines to efficiently perform *Q*-boosting of nanoresonators and metasurfaces pave the way to achieve large conversion efficiency in finite-size nanophotonic system by dynamically increasing the *Q*-factor.

## Supplementary Material

Supplementary Material Details
