# Supplementary material for: Enhancing second harmonic generation by Q-boosting lossless cavities beyond the time bandwidth limit
Source: Nanophotonics. 2024 Jan 2;13(1):1–8. doi: 10.1515/nanoph-2023-0389 (PMC11501120; doi:10.1515/nanoph-2023-0389)
Supplement: Supplementary file 1 — Supplementary Material Details [file j_nanoph-2023-0389_suppl_001.pdf]

Supporting Information

Paolo Franceschini, Andrea Tognazzi*, Anna M. Chernyak, Alexander I. Musorin, Alfonso C. Cino, Andrey A. Fedyanin, and Costantino De Angelis

Enhancing second harmonic generation by Q-boosting lossless cavities beyond the time bandwidth limit

S1 Coupled-Mode Theory (CMT)

Here, we detail CMT-based model we adopted to describe second harmonic (SH) generation in a doubly-resonant cavity, which supports modes at both fundamental frequency (FF), ω_1, and SH frequency, $\omega_2 = 2\,\omega_1$, with time-dependent amplitudes $a_1(t)$ and $a_2(t)$, respectively. The cavity is excited by an external source $s_1^+(t)$, at frequency $\omega_p = \omega_1$ (resonant excitation). In general, the set of equations describing SH generation in a doubly-resonant cavity is [1, 2]:

$$\begin{cases} \dfrac{\mathrm{d}}{\mathrm{d}t} a_1(t) = \omega_1 \left(i - \dfrac{1}{2Q_1} \right) a_1(t) - i\omega_1\,\beta_1 a_1^*(t)\,a_2(t) + \sqrt{\dfrac{\omega_1}{Q_1}}\,s_1^+(t) \\ \dfrac{\mathrm{d}}{\mathrm{d}t} a_2(t) = \omega_2 \left(i - \dfrac{1}{2Q_2} \right) a_2(t) - i\omega_2\beta_2 a_1^2(t) \\ \quad s_1^-(t) = -s_1^+(t) + \sqrt{\dfrac{\omega_1}{Q_1}}\,a_1(t) \\ \quad s_2^-(t) = \sqrt{\dfrac{\omega_2}{Q_2}}\,a_2(t) \end{cases} , \tag{S1}$$

where Q_1 and Q_2 are the Q-factors of modes 1 (FF) and 2 (SH), β_1 and β_2 are the internal cavity coupling coefficients, which are responsible for pump depletion and SH conversion, respectively, and they are related to the overlap integral between the FF and SH modes [2, 3]. As pointed out in [2], we set $\beta_2 = \beta_1/2$ to fulfill the conservation energy constraint. The terms $s_1^-(t)$ and $s_2^-(t)$ represent the outgoing waves at ω_1 and ω_2, respectively.

The results reported in Sec. 2.1 of the paper have been obtained by considering constant Q_1 and Q_2 terms in Equation S1 for both CW and pulsed input cases. The results reported in Sec. 2.2 of the manuscript have been obtained by considering time-dependent Q_1 (as in Equation 2 of the paper) and constant Q_2 term in Equation S1.

In Sections S2, S3 we provide additional details about the two regimes we analyzed: the continuous-wave (Section S2) and pulsed (Section S3) input source. In particular, we provide a summary of the quantities and their corresponding units of measurements.

S2 Continuous Wave Input Source

S2.1 Quantities and Units

In the continuous-wave (CW) input source condition (*i.e.*, $s_1^+(t) = s_1^+ \cdot e^{i\omega_p t}$), the units of the quantities involved (see Table S1) are defined consistently with [2, 4]. In particular, $s_1^+(t)$ is normalized such that $\left|s_1^+\right|^2$ is the power carried by the incident wave and $|a_k|^2$ ($k = 1, 2$) is normalized to the energy.

Quantity	Units
$a_1(t)$, $a_2(t)$	$\sqrt{\text{J}}$
$s_1^+(t)$, $s_1^-(t)$, $s_2^-(t)$	$\sqrt{\text{W}}$
β_1, β_2	$1/\sqrt{\text{J}}$

Tab. S1: Summary of the quantities and corresponding units for the CW case.

The results reported in Figure 1a (red curve) of the manuscript consists in the steady state solution of Equation S1. As in [2], we first assumed

$$a_k(t) = \tilde{a}_k(t)\, e^{i\omega_k t}, \quad s_1^+(t) = s_1^+\, e^{i\omega_1 t}, \quad \text{and} \quad s_k^-(t) = \tilde{s}_k^-(t)\, e^{i\omega_k t}. \tag{S2}$$

Under this hypothesis, the system in Equation S1 becomes

$$\begin{cases} \dfrac{\mathrm{d}}{\mathrm{d}t}\tilde{a}_1(t) = -\dfrac{\omega_1}{2Q_1}\,\tilde{a}_1(t) - i\omega_1\,\beta_1\tilde{a}_1^*(t)\,\tilde{a}_2(t) + \sqrt{\dfrac{\omega_1}{Q_1}}\,s_1^+ \\ \dfrac{\mathrm{d}}{\mathrm{d}t}\tilde{a}_2(t) = -\dfrac{\omega_2}{2Q_2}\,a_2(t) - i\omega_2\beta_2\tilde{a}_1^2(t) \\ \tilde{s}_1^-(t) = -s_1^+ + \sqrt{\dfrac{\omega_1}{Q_1}}\,\tilde{a}_1(t) \\ \tilde{s}_2^-(t) = \sqrt{\dfrac{\omega_2}{Q_2}}\,\tilde{a}_2(t) \end{cases} . \tag{S3}$$

We numerically solved the system in Equation S3, endowed by the initial conditions $\tilde{a}_1(0) = 0$ and $\tilde{a}_2(0) = 0$, to evaluate the steady state values

$$s_{1,ss}^- = \lim_{t\to+\infty} \tilde{s}_1^-(t) \qquad \text{and} \qquad s_{2,ss}^- = \lim_{t\to+\infty} \tilde{s}_2^-(t) \tag{S4}$$

In the CW case, the power conservation condition ($P_1^+ = P_1^- + P_2^-$) at steady state (ss) takes the form

$$\left|s_1^+\right|^2 = \left|s_{1,ss}^-\right|^2 + \left|s_{2,ss}^-\right|^2 \tag{S5}$$

S2.2 Complete Conversion Condition

As described in [2], the condition for complete second harmonic (SH) conversion efficiency at steady state (ss) is given by the following relation between the cavity parameters

$$\left|s_{2,ss}^-\right|^2 = \left|s_1^+\right|^2 = \frac{\omega_1}{2\,|\beta_1|^2\,Q_1^2\,Q_2}. \tag{S6}$$

S3 Pulsed Input Source

S3.1 Quantities and Units

For a pulsed input source as the one in Equation 2 of the paper, the units of the quantities involved (see Table S2) are defined consistently with [5, 6]. In particular, $s_1^+(t)$ is normalized such that $V_1^+ \int_{-\infty}^{+\infty} \left|s_1^+(t)\right|^2 \mathrm{d}t$ is the energy carried by the incident wave and $U_k = \int_{-\infty}^{+\infty} |a_k(t)|^2 \,\mathrm{d}t$ $(k = 1, 2)$ is the energy stored in the k-th mode. The pulse peak power is defined as $P_k^\pm = V_k^\pm / \Delta t_k^\pm$, where $\Delta t_k^\pm$ is the temporal duration of the pulse entering (or leaving) the cavity (see Sec. S3.2 for more details).

Quantity	Units
$\tilde{a}_1$, $\tilde{a}_2$	$\sqrt{\mathrm{J}}$
$\tilde{s}_1^+$, $\tilde{s}_1^-$, $\tilde{s}_2^-$	$\sqrt{\mathrm{W}}$
s_0	$\sqrt{\mathrm{J}}$
β_1, β_2	$\sqrt{\mathrm{fs/J}}$

Tab. S2: Summary of the quantities and corresponding units for the pulsed case.

Regarding the pulsed input source, we assume that

$$a_1(t) = \tilde{a}_1(t)\, e^{i\omega_1 t}, \quad a_2(t) = \tilde{a}_2(t)\, e^{i2\omega_1 t}, \quad s_1^\pm(t) = \tilde{s}_1^\pm(t)\, e^{i\omega_1 t}, \quad \text{and} \quad s_2^-(t) = \tilde{s}_2^-(t)\, e^{i2\omega_1 t}; \tag{S7}$$

therefore, the system in Equation S1 takes the form

$$\begin{cases} \dfrac{\mathrm{d}}{\mathrm{d}t}\tilde{a}_1(t) = -\dfrac{\omega_1}{2Q_1}\tilde{a}_1(t) - i\omega_1\,\beta_1\tilde{a}_1^*(t)\,\tilde{a}_2(t) + \sqrt{\dfrac{\omega_1}{Q_1}}\,\tilde{s}_1^+(t) \\ \dfrac{\mathrm{d}}{\mathrm{d}t}\tilde{a}_2(t) = -\dfrac{\omega_2}{2Q_2}\tilde{a}_2(t) - i\omega_2\beta_2\tilde{a}_1^2(t) \\ \tilde{s}_1^-(t) = -\tilde{s}_1^+(t) + \sqrt{\dfrac{\omega_1}{Q_1}}\,\tilde{a}_1(t) \\ \tilde{s}_2^-(t) = \sqrt{\dfrac{\omega_2}{Q_2}}\,\tilde{a}_2(t) \end{cases} . \tag{S8}$$

We numerically solved the system in Equation S8 endowed by the following initial conditions for the mode amplitudes

$$\lim_{t\to-\infty} \tilde{a}_1(t) = 0 \qquad \text{and} \qquad \lim_{t\to-\infty} \tilde{a}_2(t) = 0. \tag{S9}$$

Moreover, we note that the settings of the numerical method (adopted to solve Equation S8) and the extrema of the time domain (t_1 and t_2 with $t_1 < t_2$) were chosen in order to fulfill the energy conservation condition (Equation S10) and to be consistent with Equation S9.

In the pulsed case, the energy conservation condition ($V_1^+ = V_1^- + V_2^-$) takes the form

$$\int_{-\infty}^{+\infty} \left|\tilde{s}_1^+(t)\right|^2 \mathrm{d}t = \int_{-\infty}^{+\infty} \left|\tilde{s}_1^-(t)\right|^2 \mathrm{d}t + \int_{-\infty}^{+\infty} \left|\tilde{s}_2^-(t)\right|^2 \mathrm{d}t. \tag{S10}$$

S3.2 Pulse Duration Calculation

The pulse peak power is defined as $P_k^{\pm} = V_k^{\pm}/\Delta t_k^{\pm}$, where $V_k^{\pm}$ is the pulse energy and $\Delta t_k^{\pm}$ is temporal duration (width of the intensity profile $\left|s_k^{\pm}(t)\right|^2$) of the pulse entering (or leaving) the cavity. In the case of the input pulse $s_1^{+}(t)$, $V_1^{+} = s_0^2$ and $\Delta t_1^{+} = \tau_p$; therefore,

$$P_1^{+} = \frac{s_0^2}{\tau_p}. \tag{S11}$$

In the case of the output pulse $s_2^{-}(t)$, the pulse energy is $V_2^{-} = \int_{-\infty}^{+\infty} \left|s_2^{-}(t)\right|^2 \mathrm{d}t$ and, due to the asymmetric temporal profile, the temporal duration is defined as $\Delta t_2^{-} = 2 \cdot \sqrt{2\ln 2} \cdot \delta t_2^{-}$, where δt_2^{-} is the standard deviation (i.e., square root of the variance) of the temporal profile $\left|s_2^{-}(t)\right|^2$ and it is calculated as [7]

$$\delta t_k^{\pm} = \sqrt{\langle t^2, \left|s_k^{\pm}(t)\right|^2\rangle - \langle t, \left|s_k^{\pm}(t)\right|^2\rangle^2}. \tag{S12}$$

In particular, in Eq. (S12),

$$\langle t^q, \left|s_k^{\pm}(t)\right|^2\rangle = \frac{\int_{-\infty}^{+\infty} t^q \left|s_k^{\pm}(t)\right|^2 \mathrm{d}t}{\int_{-\infty}^{+\infty} \left|s_k^{\pm}(t)\right|^2 \mathrm{d}t} = \frac{1}{V_k^{\pm}} \int_{-\infty}^{+\infty} t^q \left|s_k^{\pm}(t)\right|^2 \mathrm{d}t \qquad (q = 1, 2 \text{ and } k = 1, 2). \tag{S13}$$

We underline that the definition in Eq. (S12) is consistent with Eq. (S11), the latter regarding the input pulse $s_1^{+}(t)$. Indeed, in the case of the input pulse $s_1^{+}(t)$, for which $V_1^{+} = s_0^2$, Eq. (S13) yields to

$$\langle t, \left|s_1^{+}(t)\right|^2\rangle = \frac{1}{s_0^2} \int_{-\infty}^{+\infty} t \left|s_1^{+}(t)\right|^2 \mathrm{d}t = \int_{-\infty}^{+\infty} t \cdot \sqrt{\frac{4\ln 2}{\pi\tau_p^2}}\, e^{-\frac{4\ln 2}{\tau_p^2}(t-\tau)^2} \mathrm{d}t = \tau \tag{S14}$$

and

$$\langle t^2, \left|s_1^{+}(t)\right|^2\rangle = \frac{1}{s_0^2} \int_{-\infty}^{+\infty} t^2 \left|s_1^{+}(t)\right|^2 \mathrm{d}t = \int_{-\infty}^{+\infty} t^2 \cdot \sqrt{\frac{4\ln 2}{\pi\tau_p^2}}\, e^{-\frac{4\ln 2}{\tau_p^2}(t-\tau)^2} \mathrm{d}t = \tau^2 + \left(\frac{\tau_p}{2\sqrt{2\ln 2}}\right)^2; \tag{S15}$$

therefore, $\delta t_1^{+} = \sqrt{\langle t^2, \left|s_1^{+}(t)\right|^2\rangle - \langle t, \left|s_1^{+}(t)\right|^2\rangle^2} = \tau_p/\left(2\sqrt{2\ln 2}\right)$ and, thus, we recover that

$$\Delta t_1^{+} = 2 \cdot \sqrt{2\ln 2} \cdot \delta t_1^{+} = \tau_p. \tag{S16}$$

S3.3 Energy and Power second harmonic conversion efficiencies

In this section, we provide additional details on the calculation of SH efficiency and we show that, for the case under analysis, the power and energy conversion efficiencies lead to the same optimal parameter estimation. In particular, we focus on the the results reported in Fig. 2a of the manuscript and we describe them in the framework of power conversion efficiency (ζ_{SH}), defined as $\zeta_{SH} = (V_2^-/\Delta t_2^-)/(s_0^2/\tau_p) = \eta_{SH} \cdot \tau_p/\Delta t_2^-$.

First, the temporal duration of the output pulse at SH was numerically retrieved from the temporal dynamics of $\left|s_2^-(t)\right|^2$ as detailed in Sec. S3.2. In particular, regarding the numerical simulations discussed in Fig. 2a of the manuscript, Fig. S1a displays the temporal width δt_2^- as a function of the delay time τ; although a variation of δt_2^- occurs, its amplitude is relatively moderate and a dip occurs close to the delay time maximizing energy conversion efficiency. Therefore, this result allows to conclude that the description in terms of SH energy conversion efficiency already provides the same information about the optimum delay time (τ^{opt}) as SH power conversion efficiency. This is confirmed by the good overlap between η_{SH} and ζ_{SH} curves displayed in Fig. S1b.

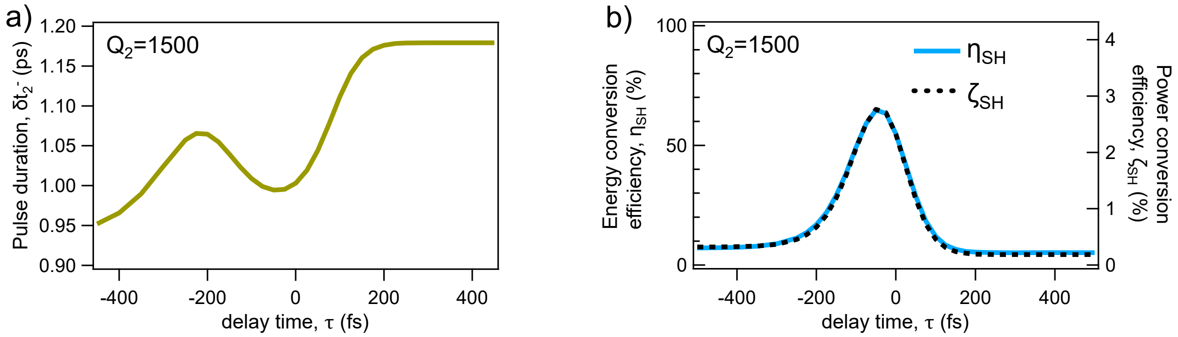

Fig. S1: (a) Pulse duration δt_2^- of the output beam at second harmonic as a function of the delay time τ. (b) Comparison between energy conversion efficiency (η_{SH}, blue solid line, left axis) and power conversion efficiency (ζ_{SH}, black dotted line, right axis) as a function of τ. The displayed quantities corresponds to the simulations reported in Fig. 2a of the main paper ($\tau_p = 100$ fs, $Q_{1L} = 55$, $\rho = 20$, and $Q_2 = 1500$).

Moreover, in order to further confirm the agreement between the two descriptions (in terms of η_{SH} and ζ_{SH}), in Fig. S2, we report the comparison between η_{SH} (at τ^{opt}) and ζ_{SH} (at τ^{opt}) as a function of the initial Q-factor of mode 1 for the three values of ρ displayed in Fig. 3a of the manuscript.

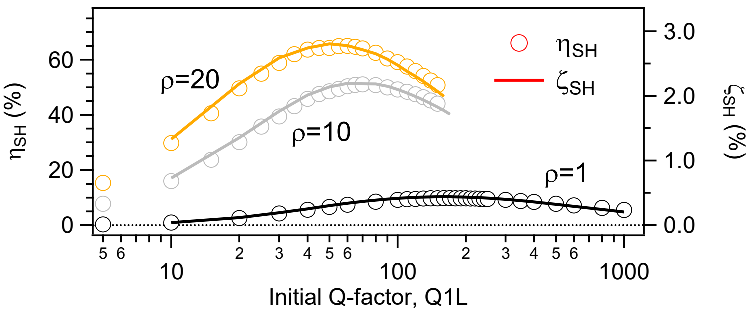

Fig. S2: Comparison between energy conversion efficiency at τ^{opt} (η_{SH}, markers, left axis) and power conversion efficiency at τ^{opt} (ζ_{SH}, solid line, right axis) as a function of initial Q-factor value Q_{1L}. The displayed quantities corresponds to the simulations reported in Fig. 3a of the paper ($\tau_p = 100$ fs and $Q_2 = 1500$).

S3.4 Additional Notes on the Static Case

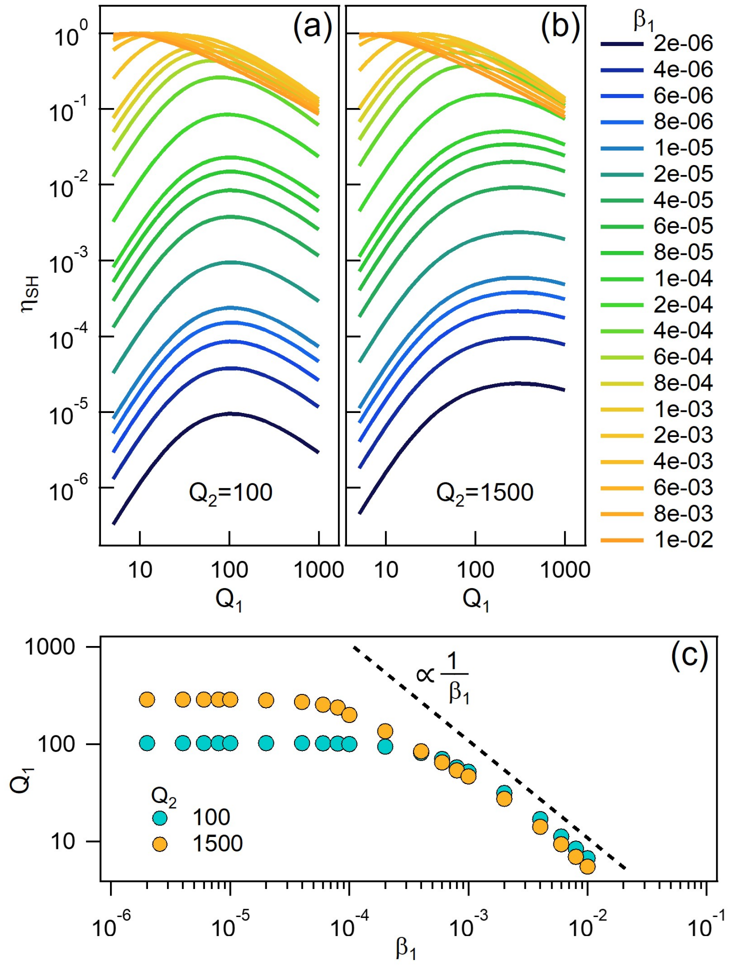

Fig. S3: (a, b) Second harmonic efficiency (η_{SH}) as a function of the Q-factor of mode at fundamental frequency (Q_1) for different values of the internal coupling parameter (β_1). The simulations have been performed with (a) $Q_2 = 100$ and (b) $Q_2 = 1500$. (c) Optimum Q_1 value as a function of β_1 retrieved from the curves in panel a and b. The dashed black line represents the set of straight lines with slope $1/\beta_1$.

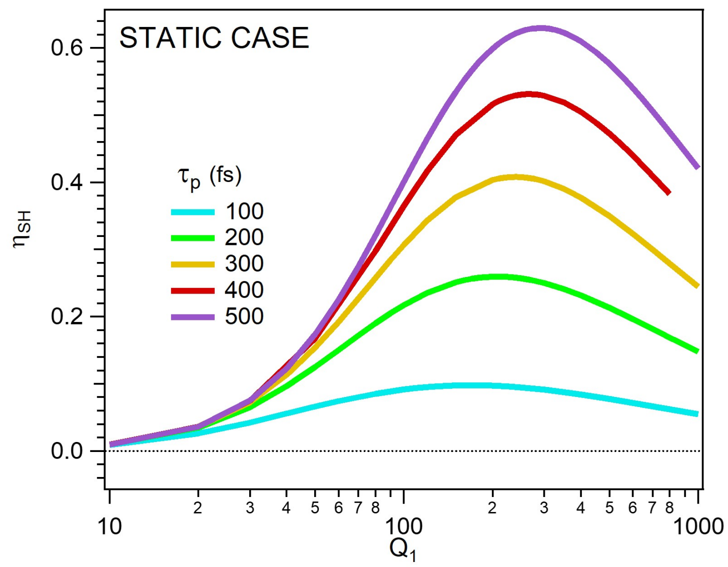

Fig. S4: Second harmonic efficiency (η_{SH}) as a function of the Q-factor of mode at fundamental frequency (Q_1) for different values of the pulse duration (τ_p). Shorter pulses have smaller efficiencies and lower optimal Q-factors.

S3.5 Optimum Delay Time

In this section we provide additional details on the dependence of η_{SH} on τ and Q_{1L}. In particular, Figure S5 shows the $\eta_{SH}(\tau, Q_{1L})$ maps for various values of ρ: 2 (panel a), 5 (panel b), 20 (panel c), and 500 (panel d). The dashed black lines highlight the dispersion curves of the optimum delay time τ^{opt}, which are summarized in Figure 2b of the paper.

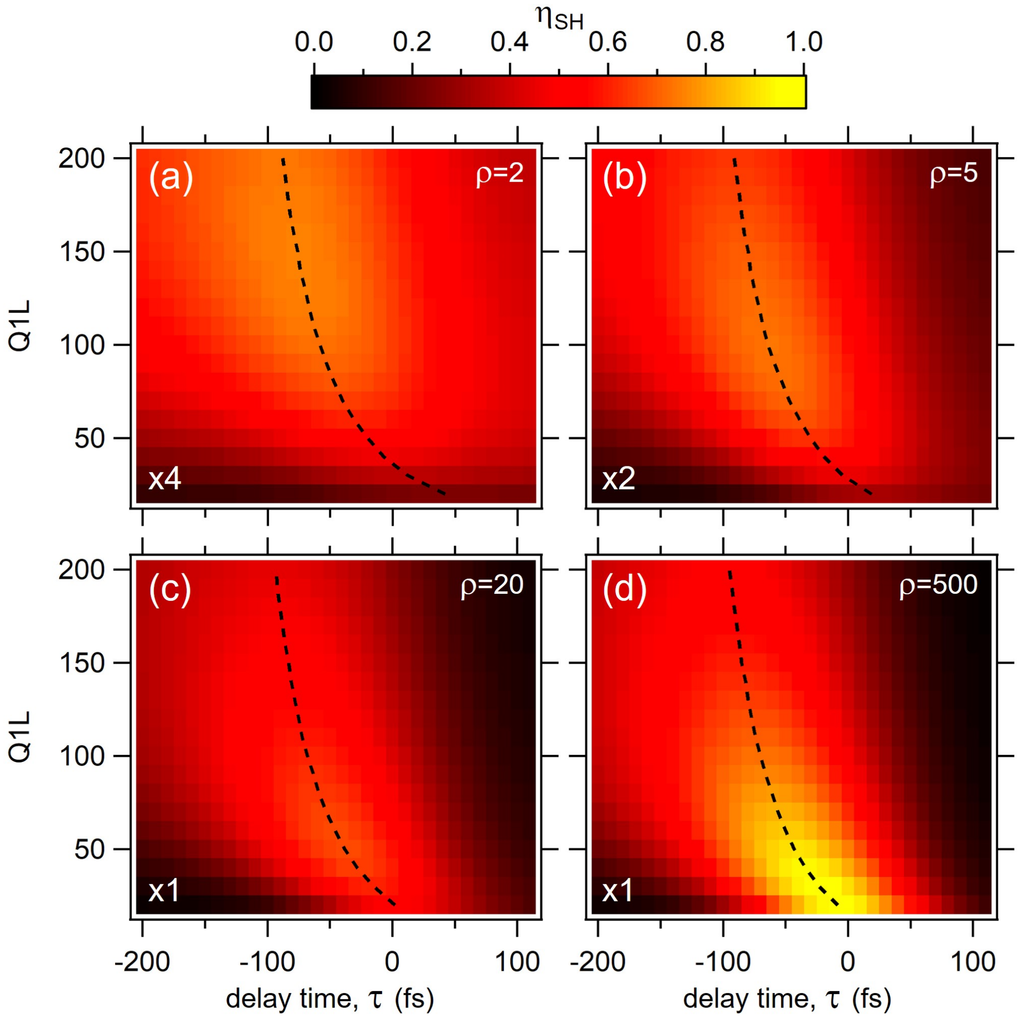

Fig. S5: Second harmonic efficiency (η_{SH}) as a function of the initial quality-factor (Q-factor) of mode at fundamental frequency (Q_{1L}) and delay time τ for different values of $\rho = Q_{1H}/Q_{1L}$, where Q_{1H} is the final Q-factor: 1 (a), 5 (b), 20 (c), and 500 (d). For a better presentation of the results, the data in panel a (b) have been multiplied by a factor $\times 4$ ($\times 2$). The dashed black line denotes the optimum delay curve dispersion (τ^{opt} vs Q_{1L}). The pulse duration is set to $\tau_p = 100$ fs.

S4 Spectral Overlap between Pulse and Mode

In this section we detail the procedure we adopted to calculate the value of the mode bandwidth allowing to optimize the spectral overlap with the control pulse. In particular, we consider normalized spectral profiles for the FF cavity mode ($\mathcal{L}_{FF}$) and external pulse ($\mathcal{G}$)

$$\mathcal{L}_{FF}(\omega) = \frac{1}{\pi} \cdot \frac{\Gamma/2}{(\Gamma/2)^2 + (\omega - \omega_1)^2} \qquad \text{and} \qquad \mathcal{G}(\omega) = \sqrt{\frac{4\ln 2}{\pi\,\Delta\omega_p^2}} \exp\left[-\frac{4\ln 2}{\Delta\omega_p^2} \cdot (\omega - \omega_1)^2\right], \tag{S17}$$

where Γ ($\Delta\omega_p$) is the full width at half maximum of the mode (pulse) spectrum and ω_1 is the central frequency. We note that

$$\int_{-\infty}^{+\infty} \mathrm{d}\omega\, \mathcal{L}_{FF}(\omega) = 1 \qquad \text{and} \qquad \int_{-\infty}^{+\infty} \mathrm{d}\omega\, \mathcal{G}(\omega) = 1. \tag{S18}$$

First, we calculate the fraction of the spectral power of $\mathcal{G}(\omega)$ within the frequency interval between $\omega_A = \omega_1 - \Delta\omega_p/2$ and $\omega_B = \omega_1 + \Delta\omega_p/2$:

$$\mathcal{G}_{AB} = \int_{\omega_A}^{\omega_B} \mathrm{d}\omega\, \mathcal{G}(\omega) = \frac{2}{\sqrt{\pi}} \int_0^{\sqrt{\ln 2}} e^{-t^2}\, \mathrm{d}t = \mathrm{erf}\left(\sqrt{\ln 2}\right) \simeq 0.760968 \tag{S19}$$

Then, we calculate the fraction of the spectral power of $\mathcal{L}_{FF}(\omega)$ within the same frequency interval

$$\mathcal{L}_{AB}(\Gamma) = \int_{\omega_A}^{\omega_B} \mathrm{d}\omega\, \mathcal{L}_{FF}(\omega) = \frac{2}{\pi} \arctan\left(\frac{\Delta\omega_p}{\Gamma}\right). \tag{S20}$$

Finally, we retrieve the value $\tilde{\Gamma}$ (see Figure S6), for which $\mathcal{G}_{AB} = \mathcal{L}_{AB}(\tilde{\Gamma})$, and we obtain $\tilde{\Gamma}/\Delta\omega_p \simeq 0.4 < 1$. Thus, $\tilde{\Gamma} < \Delta\omega_p$.

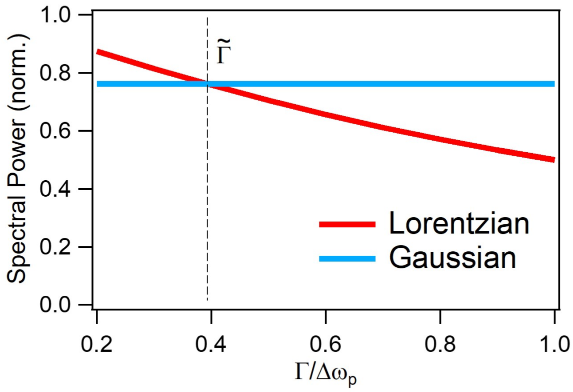

Fig. S6: $\mathcal{L}_{AB}$ as a function of $\Gamma/\Delta\omega_p$ (red curve), calculated from Eq. S20. The blue curve denotes the value $\mathcal{G}_{AB}$. The vertical dashed line highlights the value $\tilde{\Gamma}$.

S5 Spectral Analysis

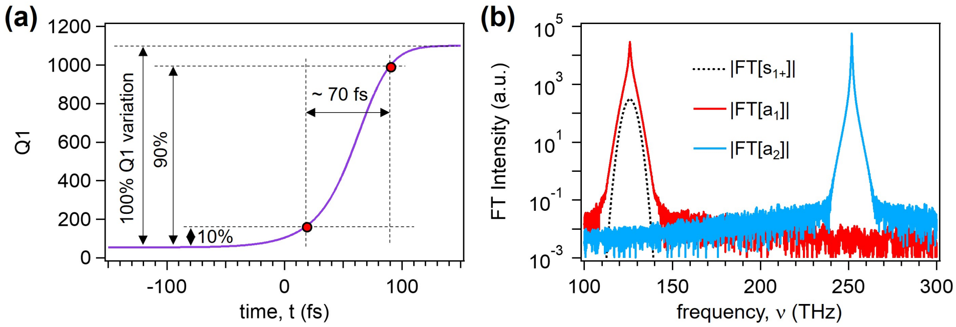

Fig. S7: (a) Temporal profile of the external coupling term Q_1 adopted in the case displayed in Figure 2a of the manuscript. (b) Fourier-Transform intensity (magnitude) of a_1, a_2, and s_1^+ obtained from the analysis of the data in Figure 2a of the manuscript. The vertical axis is in logarithmic scale. The horizontal axis is in natural frequency $\nu = \omega/(2\pi)$.

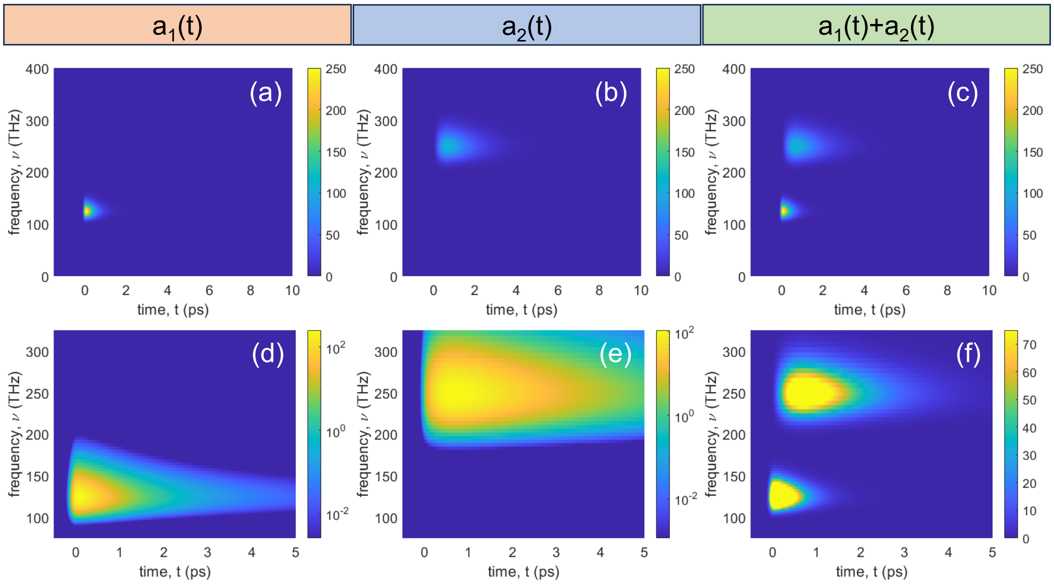

Fig. S8: Continuous wavelet (CW) analysis of the data reported in Figure 2a of the manuscript: overview (a, b, c) and zoom with saturated colorscale (d, e, f). Left panels (a, d) display CW analysis performed on $a_1(t)$, central panels (b, e) display CWA performed on $a_2(t)$, and right panels (c, f) display CW analysis performed on $a_1(t) + a_2(t)$. The maps display the squared modulus of the magnitude as a function of time (t, horizontal axis) and natural frequency (ν, vertical axis). In panels d and e the colorbar is in logarithmic scale.

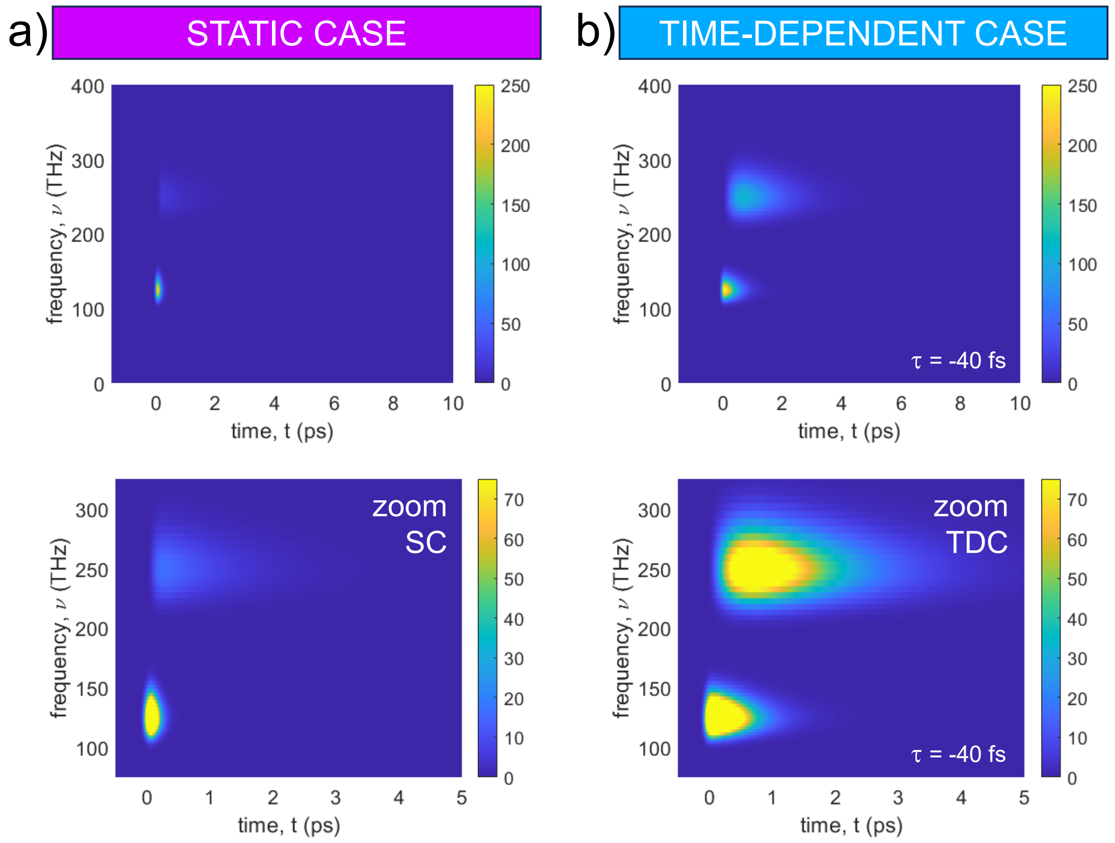

Fig. S9: Comparison between continuous wavelet (CW) analysis output for static (a, left panels, $\rho = 1$) and time-dependent (b, right panels, $\rho = 20$) cases. CW analysis have been performed on $a_1(t) + a_2(t)$ calculated with $Q_{1L} = 55$, $Q_2 = 1500$, and $\tau_p = 100$ fs. The maps display the squared modulus of the magnitude as a function of time (t, horizontal axis) and natural frequency (ν, vertical axis). Bottom panels display a zoomed view with saturated colorscale of the corresponding upper panels. SC: static case; TDC: time-dependent case. Top panel b corresponds to Figure S8c.

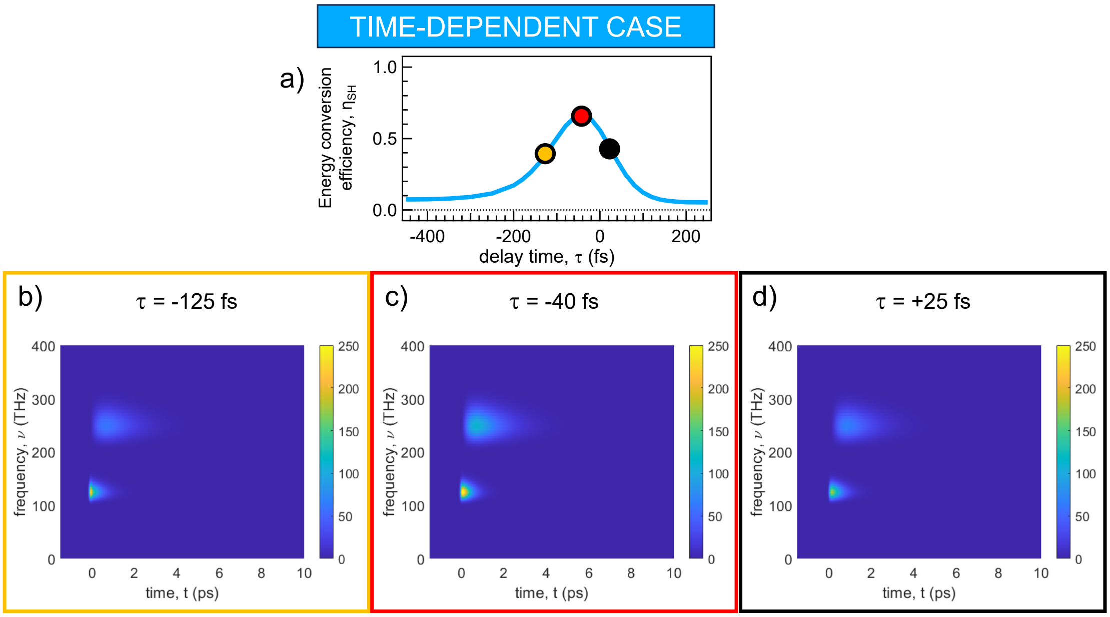

Fig. S10: Continuous wavelet (CW) analysis output at different delay time τ values. (a) Energy conversion efficiency profile as a function of delay time τ (as in the inset of Figure 2a of the manuscript). (b-d) CW analysis performed on $a_1(t) + a_2(t)$ calculated with $Q_{1L} = 55$, $\rho = 20$, $Q_2 = 1500$, and $\tau_p = 100$ fs for the following τ values: $\tau = -125$ fs (b, yellow marker in panel a), $\tau = -40$ fs(c, red marker in panel a), and $\tau = +25$ fs (d, black marker in panel a). The maps display the squared modulus of the magnitude as a function of time (t, horizontal axis) and natural frequency (ν, vertical axis). Panel c corresponds to Figure S8c.

References

[1] Zin Lin et al. "Cavity-enhanced second-harmonic generation via nonlinear-overlap optimization". In: *Optica* 3.3 (Mar. 2016), p. 233. DOI: 10.1364/optica.3.000233.

[2] Alejandro Rodriguez et al. "$\chi^{(2)}$ and $\chi^{(3)}$ harmonic generation at a critical power in inhomogeneous doubly resonant cavities". In: *Optics Express* 15.12 (May 2007), p. 7303. DOI: 10.1364/oe.15.007303.

[3] Simone Zanotti et al. "Doubly-Resonant Photonic Crystal Cavities for Efficient Second-Harmonic Generation in III–V Semiconductors". In: *Nanomaterials* 11.3 (Feb. 2021), p. 605. DOI: 10.3390/nano11030605.

[4] Hermann A. Haus. *Waves and fields in optoelectronics*. Prentice-Hall, 1984, p. 402. ISBN: 0139460535.

[5] Pavel A. Shafirin et al. "Nonlinear response of Q-boosting metasurfaces beyond the time-bandwidth limit". In: *Nanophotonics* 11.17 (May 2022), pp. 4053–4061. DOI: 10.1515/nanoph-2022-0082.

[6] Maxim R. Shcherbakov, Pavel Shafirin, and Gennady Shvets. "Overcoming the efficiency-bandwidth tradeoff for optical harmonics generation using nonlinear time-variant resonators". In: *Physical Review A* 100.6 (Dec. 2019), p. 063847. DOI: 10.1103/physreva.100.063847.

[7] N.A. Weiss, P.T. Holmes, and M. Hardy. *A Course in Probability*. Pearson Addison Wesley, 2005. ISBN: 9780321189547. URL: https://books.google.it/books?id=p-rwJAAACAAJ.
